# Sentinel node detection in muscle-invasive urothelial bladder cancer is feasible after neoadjuvant chemotherapy in all pT stages, a prospective multicenter report

**DOI:** 10.1007/s00345-016-1952-x

**Published:** 2016-10-13

**Authors:** Robert Rosenblatt, Markus Johansson, Farhood Alamdari, Alexander Sidiki, Benny Holmström, Johan Hansson, Janos Vasko, Per Marits, Susanne Gabrielsson, Katrine Riklund, Ola Winqvist, Amir Sherif

**Affiliations:** 1 Department of Urology, Stockholm South General Hospital, Karolinska Institutet, Stockholm, Sweden; 2 Department of Urology, Sundsvall Hospital, Sundsvall, Sweden; 3 Department of Surgical and Perioperative Sciences, Urology and Andrology, Umeå University, 901 85 Umeå, Sweden; 4 Department of Urology, Västmanland Hospital, Västerås, Sweden; 5 Department of Urology, Länssjukhuset Ryhov, Jönköping, Sweden; 6 Department of Urology, Akademiska University Hospital, Uppsala, Sweden; 7 Centre for Research and Development, Faculty of Medicine, Uppsala University, County Council of Gävleborg, Uppsala, Sweden; 8 Department of Medical Biosciences, Pathology, Umeå University, Umeå, Sweden; 9 Department of Medicine, Unit for Immunology and Allergy, Karolinska Institutet, Stockholm, Sweden; 10 Department of Radiation Sciences, Umeå University, Umeå, Sweden

**Keywords:** Urinary bladder neoplasms, Neoadjuvant therapy, Cisplatin, Sentinel lymph node biopsy, Cystectomy, Immunotherapy

## Abstract

**Purpose:**

To determine whether sentinel node detection (SNd) in muscle-invasive urothelial bladder cancer (MIBC) can be performed in patients undergoing neoadjuvant chemotherapy (NAC) and determine whether SNd is feasible in all pT stages, including pT0.

**Background:**

Previous published series of SNd in MIBC have not included patients undergoing NAC, and systematic reports of pT0 patients w/wo NAC were absent. Translational immunological tumor research on MIBC focusing on SNd, in the era of NAC, requires technical feasibility. Additionally, SNd in MIBC requests further evaluations as a method for nodal staging.

**Materials and methods:**

Ninety-nine patients with suspected urothelial MIBC were prospectively selected from six urological centers. After TUR-B and primary staging, 65 MIBC patients qualified for radical cystectomy. Precystectomy staging was cT2a-T4aN0M0, including 47 NAC patients and 18 chemo-naïve patients. All 65 patients underwent intraoperative SNd by peritumoral injection of 80 Mbq Technetium and Geiger probe detection. Postcystectomy staging was pT0-T4aN0-N2M0. SNs were defined by two calculations, SNdef1 and SNdef2.

**Results:**

Totally 1063 lymph nodes were removed (total SNs; 222–227). NAC patients with pT0 (*n* = 24) displayed a true positive detection in 91.7 % by either SNdef, with a median of 3.0 SNs. NACpT >0 patients had a true positive detection in 87 % (SNdef1) and 91.3 % (SNdef2). In a univariate analysis, patient group neither NAC nor tumor downstaging influenced detection rates, regardless of SN definition. In total eight patients, 4/22 metastatic nodes were SNs while 18/22 were non-SNs.

**Conclusions:**

Sentinel node detection in MIBC is feasible also in NAC patients, regardless of pT stage. SNd played no role in nodal staging.

## Background

Muscle-invasive bladder cancer (MIBC) accounts for 80 % of the mortality in bladder cancer [1]. Fifty percentage of MIBC patients die within 5 years from diagnosis [2]. Radical cystectomy (RC) and pelvic lymph node dissection are standard treatment, w/wo neoadjuvant chemotherapy (NAC). Evidence suggests that cisplatin combination NAC improves 5-year survival [3, 4] and should be offered to all medically fit urothelial MIBC patients [5]. The extent of lymph node dissection in RC is still being debated [6]. When the concept of sentinel node detection (SNd) was originally introduced in RC as an explorative procedure, the primary goal was to improve identification of lymph nodal metastases [7, 8]. SNd in MIBC has evolved into different endeavors to perform translational research in the fields of tumor biology and immunology [9, 10]. Previous studies on SNd have not included NAC patients [7, 8, 11]. NAC usage in MIBC generates an increasing number of completely downstaged (CD) tumors (pT0N0M0) [12]. Hypothetically, the absence of visible tumor might impair the possibility of performing SNd. NAC treatment might also cause local inflammation in the bladder wall, leading to abrogated lymph flow in local lymph vessels, impairing successful SNds. No previous studies have reported systematically of SNd in NAC-treated patients or in pT0 patients w/wo NAC.

### Materials/patients

**Fig. 1:**
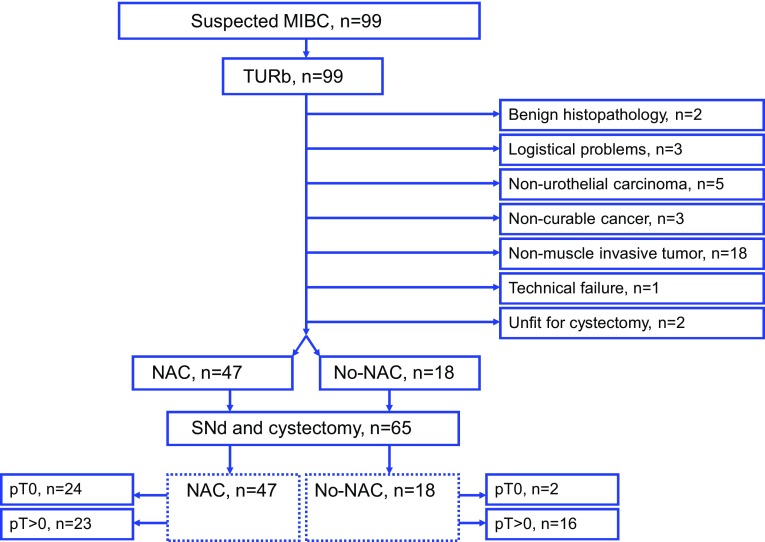
Flowchart for all included patients (*n* = 99). Patients with suspected MIBC were included prior to TUR-B. Patients without urothelial MIBC or for other reasons not considered being eligible for inclusion did not proceed to second inclusion. Totally 65 patients from the original prospective cohort fulfilled requested secondary inclusion criteria and were reincluded for SNd and RC. The flowchart also describes the outcome on pT stages over all cystectomized patients, stratified over NAC patients resp. chemo-naïve patients (No-NAC)

This non-randomized prospective trial was initiated in 2013; totally 99 patients with suspected urothelial MIBC from six Swedish urological centers were offered accrual and none declined. The patients were included stepwise, first inclusion prior to TUR-B when also blood, urine, tumor and macroscopically healthy bladder specimens were obtained for forthcoming translational research. After primary inclusion, 34 patients were aborted, the majority due to non-MIBC (exclusion data see Fig. 1). Three MIBC patients undergoing SNd were originally upstaged after undergoing second resection for first-time high-risk NMIBC (T1). The rest of the MIBC cohort undergoing SNd (*n* = 62) was primary muscle-invasive patients. The exclusion criterion for primary inclusion was previous BCG therapy. Exclusion criteria for SNd with RC were NMIBC, non-urothelial cancer, benign pathology and non-curable MIBC.

Totally 65 patients were reincluded for the second part, after being staged as urothelial MIBC; cT2-cT4aN0M0 (Fig. 1). In December 2015, the enrollment closed.

### Statistics

A univariate analysis of covariance (ANCOVA) for selected covariates was performed, followed by a multivariate ANCOVA included variables from the univariate analysis with a *p* < 0.05. The model assumption for the final multivariate model was checked using a Q–Q plot. Statistical analyses were performed in SPSS 23.

## Methods

SNd was accomplished by transurethral injection of 1 ml of intended technetium 80 MBq (Nanocoll^®^, GE Healthcare, Milan, Italy), divided and deposited at four positions near the primary bladder tumor or around the resection scar if no residual tumor. Care was taken to solely inject into the detrusor muscle without perforation. The procedure was followed by direct RC. After removal of main specimen, SNd and nodal dissection were performed. The intended areas of nodal dissection were the obturator fossae bilaterally and along the iliac arteries up to iliac bifurcations. The SNd was completed by measuring radioactivity with a handheld Geiger meter over specifically dissected nodes. Counts per minute (CPM) were recorded for both SNs and non-SNs. Harvested lymph nodes were divided; one half for routine pathology and one for immunological analysis. Charts and anatomical maps were drawn, showing node-specific CPM, anatomical positions of obtained nodes and extracted tissues possibly harboring lymph nodes.

### Definitions

Two definitions for SN were applied: SN definition-1 (SNdef1), a lymph node of ≥10 CPM detected and SN definition-2 (SNdef2), a lymph node with ≥10 % of the hottest node in each patient. The latter equals the SN with greatest CPM in a particular detection [13]. The pathology report defined harvested nodes as true lymph nodes w/wo metastasis. If a radioactive specimen showed no lymphatic tissue, it was labeled false positive (FP). If a piece of radioactive nodal tissue consisted of >1 lymph node, the CPM result was divided by the number of nodes in that specific specimen.

In each patient, we recorded the total number of harvested true lymph nodes, number of true SNs (SNdef1 and SNdef2), number of FP SNs, i.e., non-lymphatic tissue fulfilling criteria of either SNdef1 or SNdef2, false negative (FN) SNs, i.e., undetected metastatic lymph nodes.

## Results

### Outcome over three subgroups

Sixty-five patients qualified for RC and SNd, whereof 47 NAC patients and 18 chemo-naïve. Twenty-four NAC-treated patients showed CD and were termed group 1. Twenty-three NAC-treated patients were pT>0, termed group 2. All chemo-naïve patients (*n* = 18) were termed group 3 including two CD patients (Table 1). For respective pT stages, see Table 2.

**Table 1:** Patient characteristics of the 65 patients with MIBC who were reincluded for SNd and RC

Sentinel node detected patients		Group 1	Group 2	Group 3
		NACDOWN	NACnoDOWN	NoNAC
No. of patients	65	24	23	18
Age (mean)	69.1	65.5	68.4	75
Age (range)	39–86	39–79	55–80	57–86
Male	47	19	19	9
Female	18	5	4	9
Clinical stage				
cT2	44	21	10	13
cT3	20	3	12	5
cT4	1	0	1	0
Nodal yield				
Total no. harvested nodes	1063	410	371	282
Mean no. harvested nodes	16.4	17.1	16.1	15.7

The table also illustrates the three groups 1, 2 and 3

**Table 2:** Pathoanatomical outcomes over the 65 patients following SNd and RC

Histopathology	Total	Group 1	Group 2	Group 3
		NACDOWN	NACnoDOWN	NoNAC
pT0N0	26	24	0	2
pTaN0	3	0	2	1
pT1N0	3	0	2	1
pT2N0	12	0	7	5
pT3N0	12	0	5	7
pT4N0	1	0	1	0
pT0N+	1	0	1	0
pTisN+	1	0	1	0
pT2N+	2	0	2	0
pT3N+	3	0	1	2
pT4N+	1	0	1	0
Total	65	24	23	18

The table also describes the final pTNM0 stages distributed over the three groups 1, 2 and 3

Totally 1063 lymph nodes were resected, mean of 16.4 nodes/patient (Table 1). A total of 222 lymph nodes were classified as SNs according to SNdef1, mean of 3.42 SNs/patient. The equivalent for SNdef 2 was 227 nodes, mean of 3.49 (Table 4).

NAC patients with pT0 (group 1) had a true positive detection in 91.7 % of the patients regardless of SNdef compared to NACpT >0 patients (group 2) with 87 % (SNdef1) and 91.3 % (SNdef2). In group 3, the equivalent was 72.3 % (SNdef1) and 94.4 % (SNdef2) (data not shown; Table 3.

**Table 3:** Distribution of totally 22 metastatic lymph nodes in the eight patients with pN+ status

Histopathology	Metastatic lymph nodes		
	Non-SN	SN	NAC
pT0N+	2	0	Yes
pTisN+	2	0	Yes
pT2N+	0	1	Yes
pT2N+	1	0	Yes
pT3N+	0	1	No
pT3N+	3	2	No
pT3N+	1	0	Yes
pT4N+	9	0	Yes
Total	18	4	–

The majority of metastatic nodes were diagnosed in non-SNs

A median of three detected SNs was seen in group 1 and 2, with both SN definitions. Group 3 showed a slightly lower mean for SNdef1 and median, and 2 for both SNdefs. There was a tendency of higher rates of FP SNs for SNdef1 (Table 4).

**Table 4:** Results of SN detection by two definitions (SNdef1 and SNdef2) in total and distributed over the three groups 1, 2 and 3

	SNdef1		SNdef2	
Number of true SNs	222		227	
True SNs/patient	Mean	Median (range)	Mean	Median (range)
Group 1	3.63	3 (0–10)	3.17	3 (0–8)
Group 2	3.7	3 (0–9)	3.7	3 (0–11)
Group 3	2.78	2 (0–11)	3.67	2 (0–11)
All patients	3.42	2 (0–11)	3.49	3 (0–11)

Total number of FP SNs	33		30	
False FP SNs/patient	Mean	Median (range)	Mean	Median (range)
Group 1	0.5	0 (0–6)	0.5	0 (0–6)
Group 2	0.48	0 (0–5)	0.43	0 (0–5)
Group 3	0.56	0 (0–3)	0.44	0 (0–3)
All patients	0.51	0 (0–6)	0.46	0 (0–6)

The outcome of FP nodes in total and per patient is also reported. The table also records stratified mean and median values of SNs

### Clinical factors and impact on SNdef1 resp. SNdef2

A general lineal model was applied to determine whether clinical factors impacted the SN yields. Age, sex, urological department/center, cT stage, NAC, CD, pT stage, group (1, 2 or 3), lymph node metastases and total number of harvested lymph nodes were first tested individually in a univariate model. For SNdef1, age (*p* = 0.03) and number of harvested lymph nodes (*p* = 0.0001) turned out as significant factors. They were evaluated in a multivariate analysis where the number of harvested nodes again showed importance for the SNd outcome (*p* = 0.0001), whereas age no longer remained an independent predictor for SNdef1 (*p* = 0.25; Table 5).

**Table 5:** Univariate and multivariate analyses of different factors possibly having impact on SN yields for both SNdef1 and SNdef2

Predictors	True sentinel nodes		False sentinel nodes	
	SNdef1	SNdef2	SNdef1	SNdef2
	*p* value	*p* value	*p* value	*p* value
Univariate				
Age	0.03	0.51	0.93	0.92
Sex	0.6	0.75	0.97	0.74
Surgical centre	0.46	0.28	0.17	0.11
cT stage	0.17	0.27	0.57	0.57
NAC	0.27	0.749	0.83	0.94
CD	0.65	0.467	0.96	0.82
Group	0.54	0.76	0.97	0.98
pT stage	0.26	0.94	0.8	0.94
Harvested lymph nodes	0.0001	0.1	0.9	0.76
pN metastasis	0.41	0.33	0.75	0.65
Multivariate				
Age	0.25	−	−	−
Harvested lymph nodes	0.0001	−	−	−

In the multivariate analysis, finally only the total amount of harvested nodes (SNs plus non-SNs) showed importance for SNd and the total SN yield

### pN status

**Fig. 2:**
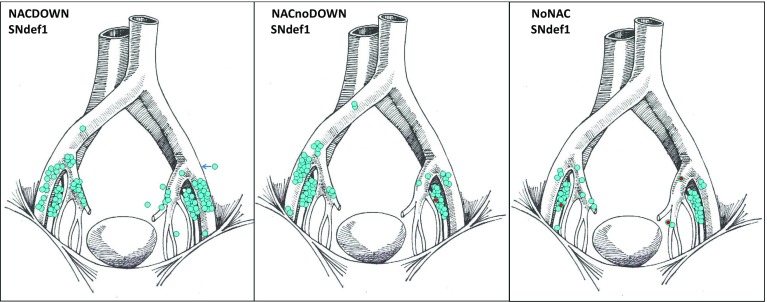
Groups 1, 2 and 3, in which group 1 represents the patients who received NAC and completely downstaged (CD) to pT0N0M0 (NACDOWN), *n* = 24, and the distribution of detected SNs by SNdef1 in the whole group. Note the blue arrow indicating a SN in the left triangle of Marseille. Group 2 was the patients who received NAC but did not reach CD (NACnoDOWN), *n* = 23, and the resp. distribution of SNs in the whole group. Finally group 3 consists of 18 patients who were chemo-naïve (NoNAC), *n* = 18. The distribution of SNs by SNdef1 was similar in all three groups. The figure also illustrates the four nodal metastases found in detected SNs of three metastatic patients (*red dots* within *blue*-marked SNs), see also Table 3. The 18 false negative metastatic nodes of six, out of totally eight patients who were pN+, are not illustrated

Eight patients had totally 22 verified nodal lymph node metastases of which 4 were identified in detected SNs (Fig. 2). Six of the metastases were found in two chemo-naïve patients. One of these patients had three metastases in non-SNs and two in SNs. In contrast, only one SN metastasis was found in the NAC group, whereas the remaining 15 positive nodes were diagnosed in non-SNs (Table 3).

## Discussion

The results of this prospective investigation show that SNd is feasible in NAC patients as well as in chemo-naïve patients, w/wo downstaging to pT0. The concept of the SN was first described by Gould et al. in the parotid gland [14] and clinically implemented by Cabanas in penile cancer [15]. One definition of the SN describes it as the initial lymph node to which the tumor drains [16]. The SN is considered being specific for a given tumor and the SN content reflecting the status of regional lymphatics. SN biopsy became well established in malignant melanoma and breast cancer. Utilized markers for detection were blue dye and radioactive tracers, yet a standardized method was lacking. How to deposit the tracers differed between investigators. The choice of radioactive tracer can also differ as, for example 99-m Tc-labeled Albures (Amersham Health, Buckinghamshire, England) with 250–500 nm particles displaying slow kinetics compared to Nanocoll with a much smaller carrier and subsequently much faster kinetics [7]. Other factors can affect the levels of accumulated tracers in a given SN; firstly, the position in the drainage order and the number of lymphatic vessels exit the individual node. Secondly, the lymph flow rate being influenced by physical exercise, medication and hydration status. In case of metastatic spread, the metastatic deposit may obstruct the entrance of lymph flow, leading to redirection of the lymphatics resulting in FN detection. This was seen in the current material where the majority of the metastasized lymph nodes were found in non-SNs. Hypothetically, chemotherapy might affect tumor lymph drainage by increasing the level of cell debris, thus obstructing lymph pathways in pN+ patients. The subgroup of lymph node metastasized patients is also too small for drawing any conclusions. The SN concept was introduced and shown feasible in MIBC in 2001 [7, 8]. Injections of tracer in MIBC-SNd are performed at four peritumoral positions of the tumor or tumor scar, preferably in non-tumorous detrusor muscle. The method was reproduced at an independent center [11], and both research groups found that >1 SN/tumor was often detected and that utilizing the handheld Geiger meter resulted in the highest SN yield. Liedberg et al detected SNs in 87 % with mean of 2.4 SN/patient. This in line with the results of the current study shows detection rates of 84.6 % (SNdef1) and 92.4 % (SNdef2) with means of 3.42 and 3.49 SNs, respectively. To define all radioactive nodes as SNs appears problematic. In melanoma, Kroon et al [13] found that defining the SN as 50 % of the hottest node yielded a FN rate of 7 %. A stepwise increase in FNR was seen for every added 10 %. In contrast to previous endeavors on SNd in MIBC, we focused on Geiger meter detected SNs and applied two different SN definitions. The 10 % rule yields a slightly larger number of SNs and a higher mean of SN/patient while decreasing the mean of FN nodes. The difference is greatest in the subgroup of chemo-naïve patients (Table 4). Regardless of SN definition, neither pT stage subgroup nor NAC affects the number of true positive SNs. SNd in postresection scars has also been feasible in penile cancer after previous removal of primary tumor [17]. The effect of NAC on SN biopsies was studied thoroughly in breast cancer showing increase in FNR [18]. However, the parallel to MIBC is not fully compatible; the use of NAC in breast cancer has increased the use of SNd on larger high-risk tumors. In contrast, our use of SNd in MIBC is not aimed at minimizing the extent of lymph node dissection or detecting nodal metastases. Due to the diversity of lymphatic drainage for MIBC in the minor pelvis, our results contravene SNd as a method for nodal staging. This is probably also due to the diversity of lymphatic drainage for MIBC in the pelvic cavity. Another challenge would be to correlate the detected SNs with a molecular signature combined with clinical factors. Our prospective series, including the present material, also forms the matrix for ongoing immunological investigations with focus on T cells, B cells, cytokines and T regulatory cells. Induction of immune responses to tumor antigens has been detected in SNs, therefore being considered a good source for harvesting tumor-specific T lymphocytes [10, 19]. These findings enabled adoptive immunotherapy utilizing autologous SN-derived T cells, both in colon cancer and in MIBC [20–22]. For performing SN-based autologous cell therapy, the technical ability to perform SNd is a primary condition, also in patients undergoing NAC, regardless of individual pathoanatomical responses. All patients in the present series underwent open cystectomy with standard SNd. We anticipate from other groups, investigations of similar character with minimal invasive surgery (MIS) as, for example, with robotically assisted radical cystectomy. SNd with MIS has shown promising results by utilizing for instance indocyanine green fluorescence imaging [23]. Limitations of the current study include the uneven distribution of NAC patients versus chemo-naïve, only 8/65 patients having nodal dissemination and a heterogeneous group of both urologic surgeons and pathologists from totally six centers.

## Conclusions

SNd in MIBC is feasible also in patients undergoing NAC, regardless of pT stage—including pT0. SNd played no role for nodal staging in the present material.
